# Community pharmacists’ skills and practice regarding dispensing fiscalized substances: a cross-sectional survey

**DOI:** 10.3389/fphar.2023.1237306

**Published:** 2023-08-31

**Authors:** Faris El-Dahiyat, Ammar Abdulrahman Jairoun, Sabaa Saleh Al-Hemyari, Moyad Shahwan, Maimona Jairoun, Sa’ed H. Zyoud, Ammar Ali Saleh Jaber, Mustfa Faisal Alkhanani, Reem Hasaballah Alhasani, Ahmed M. Ashour, Fahad S Alshehri, Nasser M. Alorfi

**Affiliations:** ^1^ Clinical Pharmacy Program, College of Pharmacy, Al Ain University, Al Ain, United Arab Emirates; ^2^ AAU Health and Biomedical Research Center, Al Ain University, Abu Dubai, United Arab Emirates; ^3^ School of Pharmaceutical Sciences, Universiti Sains Malaysia, Penang, Malaysia; ^4^ Health and Safety Department, Dubai, United Arab Emirates; ^5^ Pharmacy Department, Emirates Health Services, Dubai, United Arab Emirates; ^6^ College of Pharmacy and Health Sciences, Ajman University, Ajman, United Arab Emirates; ^7^ Centre of Medical and Bio-allied Health Sciences Research, Ajman University, Ajman, United Arab Emirates; ^8^ Department of Clinical and Community Pharmacy, College of Medicine and Health Sciences, An-Najah National University, Nablus, Palestine; ^9^ Clinical Research Centre, An-Najah National University Hospital, Nablus, Palestine; ^10^ Department of Clinical Pharmacy and Pharmacotherapeutics, Dubai Pharmacy College for Girls, Al MizharDubai, United Arab Emirates; ^11^ Biology Department, College of Sciences, University of Hafr Al Batin, Hafr Al Batin, Saudi Arabia; ^12^ Department of Biology, College of applied science, Umm Al-Qura University, Makkah, Saudi Arabia; ^13^ Department of Pharmacology and Toxicology, College of Pharmacy, Umm Al-Qura University, Makkah, Saudi Arabia

**Keywords:** numbering: continuous knowledge, practice, tramadol, community pharmacy, fiscalized substances

## Abstract

**Background:** The use of drugs containing fiscalized substances is essential in different medical areas, including pain management, obstetric emergencies, and the treatment of mental disorders. However, due to their potential for abuse and negative health effects, the dispensing of these substances demands pharmacists with the requisite skills and practice.

**Objective:** This study assesses the skills and practices of pharmacy personnel in the United Arab Emirates (UAE) regarding the dispensing of tramadol, a medication containing fiscalized substances, in community pharmacies.

**Methodology:** A cross-sectional study was conducted. Community Pharmacies were chosen via random sampling, and seven well-trained final year pharmacy students visited them and conducted face-to-face interviews. The survey tool covered items highlighting the demographic data of the subjects, and items on the practice and skills regarding dispensing the fiscalized substances. The content validity ratio values of all tool questions were more than 0.78, suggesting acceptable validity and the Cronbach’s α of 0.75 showed as acceptable internal reliability. The primary outcome measures of interest were the skills and practice regarding dispensing Fiscalized substances.

**Results:** A total of 612 pharmacists were recruited in the study. The average practice score was 80%. There was a statistically significant association (*p* < 0.05) between practices about dispensing fiscalized substances and gender, age group, pharmacy type, work experience, university of graduation, and receiving training on epilepsy and antiepileptic drugs.

**Conclusion:** The results implied that competency and experience are vital factors for the dispensing of tramadol. Contextually, the majority of the pharmacists evidently have the requisite competencies to provide high-quality and proper medical care, with regards to dispensing tramadol, which will minimize drug abuse and medication errors, and assist outpatients to manage their drugs containing fiscalized substances.

## 1 Introduction

The use of drugs containing fiscalized substances, such as benzodiazepines, tricyclic antidepressants, anxiolytics, antipsychotics, and opioid analgesics, is essential in various medical fields, including pain management, obstetric emergencies, and the treatment of mental disorders such as drug dependence, psychiatry, and neurology (Alshehri, 2023). However, due to their potential for abuse and negative health effects such as dependency syndrome, these substances are subject to international drug control treaties (World Health Organization WHO, 2012). Drugs that contain fiscalized substances carry a significant risk that the patient will develop a dependency on the medication or some form of substance use disorder (Preuss et al., 2019; Alsaab et al., 2020). Consequently, drug abuse disorders have become a prevalent issue, with several factors contributing to this problem. One reason is the practice of self-medication, often due to the increased availability of these drugs. Additionally, some individuals believe that these drugs are safer than illicit drugs, contributing to their misuse (Preuss et al., 2019). Furthermore, there are currently flaws in the monitoring and regulation of prescription procedures. For instance, it is widespread practice to administer various non-over-the-counter drugs, including those containing restricted substances, without a valid medical prescription, even in situations in which such as prescription is mandated (Alshammari et al., 2017).

Dispensing fiscalized drugs without verifying the patient’slegitimate medical prescription can lead to drug abuse, dependency, overdose, and even mortality (Alshammari et al., 2017). Based on various surveys, it has been found that amitriptyline (91.4%), tramadol (90%), and trazodone (60%) are among the most commonly fiscalized pharmaceuticals available at pharmacies and drug stores. Furthermore, approximately 60% of these establishments lack qualified personnel on their pharmacy staff. This deficiency in education and training may lead to errors in medication usage and inadequacies in the proper distribution of fiscalized pharmaceuticals (Ceballos et al., 2018).

For patients receiving pharmaceutical care, the pharmacists providing this care must possess the necessary competencies, positive attitudes, knowledge, and abilities (Alhazmi et al., 2022). Pharmacists play a critical role in ensuring drug usage accuracy, minimizing medication errors, and helping outpatients manage their pharmacotherapy effectively (Althomali et al., 2022). However, it is especially vital to improve their skills in administering medications containing fiscalized compounds, such as prohibited narcotics, to ensure the safe and responsible use of these substances. This improvement is necessary to promote teamwork and achieve optimal drug management outcomes (Walters et al., 2012; Buxton and De Muth, 2013; Wheeler et al., 2013; Kouladjian et al., 2016; Palmer et al., 2017; Cheema et al., 2018).

Ongoing training for pharmacy staff has been recognized as an essential tool to enhance their skills and promote the responsible use of pharmaceuticals, including opioids, antidepressants, and anxiolytics, while preventing their misuse and abuse (Pervanas et al., 2016). This training can focus on proper prescribing practices, monitoring signs of addiction or misuse, and ensuring that these medications are used only for legitimate medical purposes (Walters et al., 2012; Buxton and De Muth, 2013; Wheeler et al., 2013; Kouladjian et al., 2016; Palmer et al., 2017).

According to the findings of some studies, the excessive workload that is frequently forced on pharmacy staff serves to reduce the time they have available to spend with each patient and reduces the degree of control they have over medical prescriptions (Caamaño et al., 2005; Jairoun et al., 2021; Jairoun et al., 2022). The competencies of the pharmacy staff are directly correlated with their ability to enhance patients’ therapeutic outcomes and quality of life (Cheema et al., 2017). Furthermore, ongoing scientific progress and the development of public health policies depend on the specific capabilities of staff in the pharmacy setting (Federation International Pharmaceutical FIP, 2012). The competencies identified by the World Health Organization (WHO) and the Pharmaceutical International Federation (FIP) encompass a range of knowledge, abilities, and attitudes that individuals must demonstrate to complete a task or perform a job effectively. In this context, the present study aimed to assess the knowledge and skills of pharmacy personnel in the United Arab Emirates regarding the dispensing of tramadol, a medication containing fiscalized substances, in community pharmacies. This study sought to identify any gaps in knowledge or deficiencies in techniques among pharmacy staff in the UAE that could lead to medication errors or misuse of this drug.

## 2 Methods and materials

### 2.1 Study setting and design

Community pharmacists’ skills and practice regarding dispensing fiscalized substances got evaluated through a cross-sectional study design. UAE Community Pharmacies were chosen via random sampling, with seven well trained pharmacy students in their final year of study visited them and conducted face-to-face interviews between October 2022 and March 2023. Prior to the face-to-face interviews, the students were deeply trained regarding how to handle the questionnaire and on the research scientific terminology. This was due to our experience previously that showed the fact that comprehensive training boosted the interviewer’s skills and minimized the number of errors that took place during the survey.

### 2.2 Research instrument development

Based on a thorough literature review, a structured questionnaire got constructed (Ceballos et al., 2020; Ceballos et al., 2021). This was modified step by step to suit the UAE context ensuring the coverage of the key research points. Then, experts in Pharmacology were consulted to give their opinions in order to make sure that the design of the questionnaire and the relevance to the research project were acceptable, Moreover, six faculty members of Medicine and Clinical Pharmacy faculty at Ajman University were then asked to evaluate the questionnaire for relevance and appropriateness as well as its content. After that, slight amendments were done to the questionnaire based on the recommendations provided, prior to pilot testing. Recommended modifications included the scientific terminology definition, the numbering of the questions and pages modifications, changing the field name (Sex) to (Gender) in the questionnaire body, merging some questions with each others, as well as ending the questionnaire at some point.

The questionnaire’s content validity was checked via utilizing Lawshe’s content validity before pilot testing (Lawshe, 1975). Pertaining to the method (Lawshe, 1975), questions that got a content validity ratio (CVR) above 0.78 were considered acceptable, but those that do not were eliminated from the tool. The CVR values of all tool questions were more than 0.78, suggesting acceptable validity. The means of those questions that had acceptable CVR values were later used in the content validity index (CVI) calculation for the final tool. The produced CVI value of 0.891 suggested that the final tool had an acceptable overall validity (Polit et al., 2007).

To assess the tool’s face validity, we performed pilot testing of 30 community pharmacists, of which their data was excluded from the final analysis, between 8 October 2022 and 12 October 2022. 25 of these pharmacists successfully completed the questionnaire. The questionnaire reliability was later evaluated up on the results of the pilot test, and the sample size of the main research was also calculated. Finally, we used Cronbach’s α to check the questionnaire’s reliability; the Cronbach’s αof 0.75 showed as acceptable internal reliability.

### 2.3 Research instrument sections

The following 4 sections comprised the survey tool.

• Part 1—8 items highlighting the demographic data of the subjects, like their gender, official position (i.e., chief pharmacist or pharmacist in charge), professional experience years, working hours per day, numbers of patients that are served/day and if they got trained on the fiscalized substances.
• Part 2—11 items on the practice and skills regarding dispensing the fiscalized substances.

### 2.4 Questionnaire scoring

We assessed Skills and Practices regarding dispensing fiscalized substances by 11-itmes. These up mentioned items got rated on a 5-points Likert scale (1 = “Never”, 2 = “Rarely”, 3 = “Sometimes”, 4 = “Often”, 5 = “Always”). The basic scores of 1–5 got calculated for every respondent by summing up the grades of the 11 items.

Good practice score of participants was revealed by calculation of a median score in order to divide the practice scores into a good practice.

• 44 was received median score of practice. That’s why, subjects who obtained a score of 44 or higher were named as having a good practice, however, those who had a lower score of below than 44 were not.

### 2.5 Sample size calculation

The main research sample size was obtained by the pilot study. The response rate of pilot questionnaires was 83%. The subjects received the question “Do you have and a good skills and experience about dispensing fiscalized substances?” Around half (60%) of the subjects answered affirmatively. The study employed a five percent alpha level, providing a 95% confidence interval (CI). In addition, the precision (D) was five per cent, the maximum 95% CI broadness was 10%. Relying on this and assuming non-response rate of around 40%, 615 subjects was set to be an appropriate sample size.

### 2.6 Target population

The sample of main research was selected based upon some criteria. Subjects had to be community pharmacists having at least 3 months of professional experience in pharmacies whether independent ones or those belonged to a chain of pharmacies registered under the Health Authority Abu Dhabi (HAAD), Ministry of Health, or the Dubai Health Authority. Subjects were excluded in case they were not registered under any of the above-mentioned health authorities or if had not yet completed 3 months of professional experience, i.e., were still under probation, or had recently achieved their qualification.

### 2.7 Sampling technique

The researchers used stratified random sampling to ensure that the study was representative. A study in 2010 revealed that 2000 community pharmacies were active professionally in the UAE (Emirates News Agency, 2010). We got the contact details of community pharmacies in the regions chosen for the study, along with their location, from the Yellow Pages and local business directories. We divided the community pharmacies that were professionally active in the UAE into groups or strata as per their location. Three groups were determined after that, namely, community pharmacies in Abu Dhabi, community pharmacies in Dubai and community pharmacies in the Northern Emirates.

After the community pharmacies selection, all relevant data covering the pharmacy’s official name, type, location, email address, and phone number, were noted into an Excel spreadsheet that served as a sampling frame. We assigned a unique ID number to each pharmacy. After that, a simple random sample selection was done on the pharmacies liste to select 615 community pharmacies. The selected community pharmacies were then sorted by their type and location.

### 2.8 Data collection

The chosen UAE community pharmacies got visited by the trained researchers between 20 October 2022 and 25 March 2023. After informing them about the aim of the study, the pharmacists at every pharmacy were asked then for their email addresses. The researchers then conducted face-to-face interviews with them using the structured tool (questionnaire).

### 2.9 Statistical analysis

To perform the analysis of the gathered data, we used SPSS Version 26. The categorical variables got summarized as percentages and frequencies, while the continuous normally distributed quantitative variables got presented as a mean standard deviation (SD). One-way ANOVA, unpaired student t-tests, and non-parametric variants were all used to clarify if there were any differences between the quantitative variables of the groups. Assessing the normality was done by Shapiro-Wilk test (with *p* > 0.05 indicating a normally distributed continuous variable) or by visual evaluation of a Normal Q-Q Plot. The factors affecting community pharmacists’ practice got determined by utilizing multivariate logistic regression models. *p*-values below 0.05Suggested statistical significance.

### 2.10 Ethical considerations

This study got approved by Ajman University’s Institutional Ethical Review Committee (P-H-S-2022-2-12). All subjects were informed regarding the aim of the survey prior to data collection, ensuring their full understanding that their consent was needed fully for completion and submission of the tool. We obtained a written informed consent from all subjects. None of the subjects’ identities was noted, and we maintained their confidentiality by all needed steps.

## 3 Results

### 3.1 Demographic characteristics of the study population

A total of six-hundred and twelve (n = 612) pharmacists were recruited in the study (Responss rate = 99.5%). Of the total, 48.2% were male and 51.8% were female. The age of the participants as follow: 308 (50.3%) aged 25–34, 223 (36.4%) aged 35–44 and 81 (13.2%) aged ≥45. About three-quarter (76%) were chain pharmacies and 24% were independent pharmacies. Assistant pharmacists constituted 42.5% of the study sample, 10.9% were chief pharmacists and 46.6% were pharmacists in charge. The work experience was detailed as follow: 260 (42.5%) < 1 year, 44 (7.2%) 1–5 years, 58 (9.5%) 6–10 years and 250 (40.8%) > 10 years. Among the participants, 16.3% graduated from local universities, 26.1% graduated from regional universities and 57.5% graduated from international universities. The majority of the study sample received training on fiscalized substances (Table 1).

**TABLE 1:** Number and percentages of the questions on demographics (n = 612).

Demographics	Groups	Frequency	Percentages (%)
**Gender**	Male	295	48.2
	Female	317	51.8
**Age group**	25–34	308	50.3
	35–44	223	36.4
	≥45	81	13.2
**Pharmacy type**	Independent pharmacy	147	24
	Chain pharmacy	465	76
**Position in the pharmacy**	Assistant pharmacist	260	42.5
	Chief pharmacist	67	10.9
	Pharmacist in charge	285	46.6
**Work experience**	<1 year	260	42.5
	1–5 years	44	7.2
	6–10 years	58	9.5
	>10 years	250	40.8
**Graduation University**	Local	100	16.3
	Regional	160	26.1
	International	352	57.5
**Trained on fiscalized substances**	No	147	24
**Study year recoding**	Yes	465	76

Abbreviations: DM, Diabetes mellitus

### 3.2 Assessment of skill and practice about dispensing fiscalized substances in the community pharmacies

In general, the overall level of skills and practices about dispensing fiscalized substances was good. The average practice score was 80% with a 95% confidence interval (CI) [80.6%, 81.9%]. The results of each question related to skills and practice about dispensing fiscalized substances was shown in Table 2.

**TABLE 2:** Number and percentages of the questions on skills and practiceaboutdispensing fiscalized substances in the community pharmacies.

Practice items	Never		Rarely		Sometimes		Often		Always	
	F	%	F	%	F	%	F	%	F	%
When dispensing tramadol, how often do you inform the patient about the following precautions and recommendations?										
1. The use of tramadol by women in their first trimester of pregnancy, who suspect they are pregnant, or who are lactating	5	0.8	15	2.5	58	9.5	350	57.2	184	30.1
2. The use of tramadol by people who drive vehicles or use heavy or high-risk equipment concerning thepossibleside effects of drowsiness or dizziness	11	1.8	25	4.1	74	12.1	294	48	208	34
3. The frequent use of tramadol may lead to addiction or dependence	10	1.6	17	2.8	46	7.5	334	54.6	205	33.5
4. The use of tramadol by patients suffering from seizures (epilepsy)	6	1	7	1.1	36	5.9	308	50.3	255	41.7
5. The use of tramadol together with another medication that has a sedative or hypnotic effect (e.g., for anxiety, depression or sleep disorders)	3	0.5	12	2.0	29	4.7	314	51.3	254	41.5
6. Alcohol should be avoided when taking tramadol	9	1.5	25	4.1	97	15.8	365	59.6	116	19
7. I offer informational material, e.g., flyers, on the rational use of drugs to manage pain	4	0.7	25	4.1	84	13.7	386	63.1	113	18.5
8. Take tramadol irrespective of meals (i.e., before, during or after eating)	19	3.1	33	5.4	117	19.1	290	47.4	153	25
9. Avoid taking tramadol over long periods	3	0.5	14	2.3	137	22.4	334	54.6	124	20.3
10. Store tramadol appropriately in the home due to the risk it poses to others	4	0.7	14	2.3	24	3.9	287	46.9	283	46.2
11. Treatment adherence and its importance	6	1	39	6.4	151	24.7	324	52.9	92	15

Abbreviations: F, Frequency; %, Percentages

Table 3 presents the distribution of practice score according to demographic factors. There was a statistically significant associations between practices about dispensing fiscalized substances and: gender (*p* = 0.007), age group (*p* = 0.008), pharmacy type (*p* = 0.012), work experience (*p* = 0.029), university of graduation (*p* < 0.001) and receiving a training on epilepsy and antiepileptic drugs (*p* < 0.001).

**TABLE 3:** Community pharmacists’ practice and skills according to demographics.

Demographics		Practicescores (11 items)			
	N	Mean (%)	95% CI		*p*-value
**Gender**					
Male	295	80.39	79.45	81.33	0.007*
Female	317	82.17	81.28	83.07	
**Age group**					
25–34	308	80.44	79.42	81.46	0.008*
35–44	223	81.73	80.75	82.71	
≥45	81	83.48	82.13	84.83	
**Pharmacy type**					
Independent Pharmacy	147	78.54	76.94	80.14	0.012*
Chain Pharmacy	465	82.19	81.51	82.87	
**Position in the Pharmacy**					
Assistant pharmacist	260	80.42	79.25	81.59	0.063
Chief pharmacist	67	81.60	80.12	83.09	
Pharmacist in charge	285	82.06	81.23	82.89	
**Work Experiences**					
<1 year	260	80.42	79.25	81.59	0.029*
1–5 Years	44	80.99	78.94	83.04	
6–10 Years	58	80.44	77.82	83.06	
>10 years	250	82.50	81.75	83.26	
**Graduation university**					
Local	100	76.73	74.75	78.70	<0.001*
Regional	160	82.73	81.38	84.07	
International	352	81.97	81.24	82.70	
**Trained on fiscalized substances**					
No	147	78.54	76.94	80.14	<0.001*
Yes	465	82.19	81.51	82.87	

*p-values <0.05 considered statistically significant, *p*-values obtained from independent *t*-test and One Way ANOVA.

### 3.3 Factors influencing the community pharmacists’ skills and practice about dispensing fiscalized substances

The results of multivariate regression analysis showed better practice about dispensing fiscalized substances were observed in chain pharmacies (OR 1.8; 95% CI 1.07–1.29), Chief pharmacists (OR 1.17; 95% CI 1.04–1.3), Pharmacists in charge (OR 1.21; 95% CI 1.09–1.34), pharmacist with more than 10 years of experience (OR 1.23; 95% CI 1.03–1.49), graduation from international universities (OR 1.36; 95% CI 1.25–1.49) and receiving a training on fiscalized substances (OR 1.18; 95% CI 1.08–1.29) (Table 4).

**TABLE 4:** Regression analysis for the factors affecting the community pharmacists; skills and practice about dispensing fiscalized substances.

	Good practice ≥44			
Demographics	OR	95% CI		*p*-value
**Gender (Ref. Male)**				
Female	1.008	0.946	1.073	0.816
**Age group (Ref. 25–34)**				
35–44	1.003	0.888	1.133	0.960
≥45	1.123	0.970	1.299	0.120
**Pharmacy type (Ref. Independent Pharmacy)**				
Chain Pharmacy	1.181	1.078	1.293	0.001*
**Position in the Pharmacy (Ref. Assistant pharmacist)**				
Chief pharmacist	1.178	1.037	1.338	0.012*
Pharmacist in charge	1.215	1.095	1.349	<0.001*
**Experiences (Ref**. < 1 year)				
1–5 Years	1.137	0.978	1.321	0.095
6–10 Years	1.105	0.926	1.319	0.269
>10 years	1.238	1.027	1.492	0.025*
**Numbers of patients served daily (Ref. Local)**				
Regional	1.208	1.090	1.339	0.06
International	1.369	1.251	1.497	0.001*
**Trained on fiscalized substances(Ref. No)**				
Yes	1.181	1.078	1.293	<0.001*

The regression used is a multivariate logistic regression model, *p < 0.05 was considered statistically significant.

**Abbreviations:** OR; odd ratio.

## 4 Discussion

The present study assessed the knowledge and skills of pharmacy personnel in the UAE regarding the dispensing of tramadol in community pharmacies. This study also identified gaps in knowledge or deficiencies in techniques among pharmacy staff in the UAE that could lead to medication errors or tramadol abuse/misuse. The evaluated practice indicators include precautions and recommendations that cover frequency of use, treatment adherence, drug storage, use in combined drug treatment, drug dispensing process, and side-effects.

The majority of the participants responded that they either often or always inform patients about the precautions and recommendations for the use of tramadol. The bulk of respondents were females, aged 25–34, worked in chain pharmacies, worked as a pharmacist in charge, had < 1-year work experience or >10 years experience, graduated from international universities, and were trained on fiscalized substances. In relating community pharmacists’ practice and skills to demographic variables, age group, gender, pharmacy type, work experiences, graduating University and training on fiscalized substances showed statistical significance (*p* < 0.05). Regression analysis showed that chain pharmacy, chief pharmacist and pharmacist in charge positions, >10 years of work experience, international patients served daily, and training on fiscalized substances significantly affected the skills and practice of community pharmacists about dispensing fiscalized substances.

This relationship established between the demographics of the respondents and dispensing practice of drugs containing fiscalized substances is anticipated due to the fact that most of the respondents graduated from international universities with pharmacy degrees, and were trained on fiscalized substances. This shows the need for proper education and training, with an emphasis on enhancing the competency of the pharmacy staff. On the other hand, a similar study (Alshammari et al., 2017) conducted in Saudi Arabia had earlier evaluated the rate and prevalence of dispensing high-risk prescription-only medications at community pharmacies in Saudi Arabia and reported an alarming rate of non-compliance with the law of pharmaceutical practice, possibly due to poor or lack of training on dispensing drugs containing fiscalized substances. Moreover, Gokcekus et al. (Gokcekus et al., 2012), assessed the dispensing practice of community pharmacists in the Turkish Republic of Northern Cyprus (TRNC) regarding rational drug use, and evaluated the quality of dispensing. The dispensing practice appeared to be inadequate in terms of GPP, as evident by the relatively low dispensing time and failure to warn patients about potential interactions. The inadequacy was due to the employees’ lack of pharmacy-based training, which further buttresses the importance of education and specialized training.

The quality of the dispensing process is another critical component. This study demonstrated that the dispensing process in drug stores and pharmacies in UAE is moderately good since 63.1% of the respondents offered informational material (e.g., flyers) on the rational use of tramadol to manage pain. Dispensing is a process that results in a patient leaving drugstores and pharmacies with a specified quantity of drugs and instructions/precautions on drug use. Hence, in the course of dispensation, the pharmacy staff should provide adequate relevant data and information to guarantee the safe and appropriate use of medication by patients (Ceballos et al., 2021). The respondents in this study affirmed that they provided information on the proper use of tramadol and moderate practice scores, possibly due to their education, training on fiscalized substances, and work experience. Thus, they are able to effectively communicate with patients.

Tramadol has a high propensity of addiction with chronic use and it is typically associated with side effects such as nausea, emesis, diaphoresis, lethargy, sedation, and xerostomia **(Beakley et al., 2015; Miotto et al., 2017).** The results showed that the majority of the participating pharmacy staff often informed patients about the precautions and recommendations associated with the use of tramadol, which could help patients to avoid or decrease safety problems resulting from tramadol misuse. This study thus confirms the assumption by most physicians that the pharmacist will inform patients in the course of dispensing **(Toklu et al., 2010; Ceballos et al., 2021).** Similarly, the literature recommends providing patients with pertinent data that can alter their habits and improve their health, such as the recommendation of non-pharmacological treatments **(Toklu et al., 2010; Ceballos et al., 2021).** In this study, the majority of the pharmacists provided recommendations on the use of tramadol in several situations such as pregnancy, when driving, in epileptic patients, with alcohol, in combination with other drugs, and its storage.

The strength of this study is relating the demographic characteristics of the respondents to dispensing practices. This way, factors that enable the proper dispensing of tramadol were established. The results of this study imply that a competent and proficient workforce is crucial for the improvement in therapeutic outcomes, patient quality of life, scientific development, and enhanced public health **(International Pharmaceutical Federation, 2012).** Proper education, training, and experience can develop competencies in the dispensing of drugs that contain fiscalized substances. It can also be insinuated that drug retail establishments are striving to meet the minimum international standard for pharmacy practices **(Buxton et al., 2015; Scahill et al., 2017).** The results also support the need for this kind of research, which favors the need for continuing education strategies. This need for continuing education strategies is supported by similar studies **(Vacca et al., 2005; Mauricio et al., 2022).** The provision of continuing education programs by means of different strategies improves the competencies of pharmacy staff, thus enhancing their dispensing of drugs with fiscalized substances. However, there were no improvements in skills and attitudes in real practice. These findings could show that pharmacy staff needs additional and continuous training/sustainability **(Mauricio et al., 2022).** Moreover, poor dispensing practices are mostly associated with establishments whose pharmacy staff lacks pharmaceutical training **(Ceballos et al., 2021).**

A possible limitation of this study is that the visits were not audio-visually recorded to minimize potential biases related to data collection and assessment. In addition, rationality indicators (average dispensing time, stock availability and adequate labelling) were not considered in the practice items. Therefore, future studies can assess these rationality indicators and their relationship to the demographic characteristics of dispensing pharmacists. Studies can also evaluate the impact of continuing education programs on improving the skills and practices of pharmacists in dispensing drugs containing fiscalized substances.

## 5 Conclusion

This study assessed the practices of pharmacy staff operating in UAE communities when dispensing drugs with fiscalized substances. The majority of the participants were found to often inform patients about the precautions and recommendations in the use of tramadol. The results showed that chain pharmacy, positions of the chief pharmacist and pharmacist in charge, >10 years of work experience, international patients served daily, and training on fiscalized substances significantly affected the skills and practice of community pharmacists with regards to dispensing fiscalized substances. This implies that competency and experience are vital factors in the dispensing of tramadol. Contextually, the majority of pharmacists evidently have the requisite competencies to provide high-quality and proper medical care, with regards to dispensing tramadol, which will minimize drug abuse and medication errors, and assist outpatients to manage their drugs containing fiscalized substances.

## Data Availability

The original contributions presented in the study are included in the article/supplementary material, further inquiries can be directed to the corresponding authors.

## References

[B1] Alhazmi M. Bajuayfir A. Cheema E. Elrggal M. Ali M. (2022). Evaluation of current community pharmacist practice in Saudi Arabia—a cross-sectional study from pharmacists’ perspective (part II). Pharmacy 10 (2), 38. 10.3390/pharmacy10020038 35314619 PMC8938769

[B2] Alsaab H. O. Altowairqi E. Alzahrani N. Alzahrani R. Alshehri F. S. Almalki A. H. (2020). Sex differences in pregabalin-seeking like behavior in a conditioned place preference paradigm. Saudi Pharm. J. 28 (12), 1749–1755. 10.1016/j.jsps.2020.11.001 33424265 PMC7783229

[B3] Alshammari T. M. Alhindi S. A. Alrashdi A. M. Benmerzouga I. Aljofan M. (2017). Pharmacy malpractice: the rate and prevalence of dispensing high-risk prescription-only medications at community pharmacies in Saudi Arabia. Saudi Pharm. J. 25 (5), 709–714. 10.1016/j.jsps.2016.10.001 28725143 PMC5506661

[B4] Alshehri F. S. (2023). Tapentadol: A review of experimental pharmacology studies, clinical trials, and recent findings. Drug Des. Devel Ther. 17, 851–861. 10.2147/DDDT.S402362 PMC10039632 36974332

[B5] Althomali A. Altowairqi A. Alghamdi A. Alotaibi M. Althubaiti A. Alqurashi A. (2022). Impact of clinical pharmacist intervention on clinical outcomes in the critical care unit, taif city, Saudi Arabia: A retrospective study. Pharmacy 10 (5), 108. 10.3390/pharmacy10050108 36136841 PMC9498917

[B6] Beakley B. D. Kaye A. M. Kaye A. D. (2015). Tramadol, pharmacology, side effects, and serotonin syndrome: A review. Pain Physician 18 (4), 395–400. 10.36076/ppj.2015/18/395 26218943

[B7] Buxton E. C. De Muth J. E. (2013). Pharmacists’ perceptions of a live continuing education program comparing distance learning versus local learning. Res. Soc. Adm. Pharm. 9 (2), 230–235. 10.1016/j.sapharm.2012.05.003 22835712

[B8] Buxton J. A. Babbitt R. M. Clegg C. A. Durley S. F. Epplen K. T. Marsden L. M. (2015). ASHP guidelines: minimum standard for ambulatory care pharmacy practice. Am. J. Health Syst. Pharm. 72 (14), 1221–1236. 10.2146/sp150005 26150573

[B9] Caamaño F. Tomé-Otero M. Takkouche B. Gestal-Otero J. J. (2005). Influence of pharmacists’ opinions on their dispensing medicines without requirement of a doctor’s prescription. Gac. Sanit. 19 (1), 9–14. 10.1157/13071811 15745663

[B10] Ceballos M. Giraldo J. A. Marيn V. H. Amariles P. (2018). Caracterización de aspectos relacionados con la utilización de los medicamentos fiscalizados en droguerías y farmacias-droguerías de Medellín y el Área Metropolitana. Rev. Univ. Ind. Santander Salud 50 (1), 27–36. 10.18273/revsal.v50n1-2018003

[B11] Ceballos M. Salazar-Ospina A. Sabater-Hernández D. Amariles P. (2020). Evaluation of the effects of a drug with fiscalized substance dispensation, health education, and pharmacovigilance continuing education program in Colombia drugstores and drugstores/pharmacies: study protocol of a multicenter, cluster-randomized controlled trial. Trials 21 (1), 1–4. 10.1186/s13063-020-04481-1 32560735 PMC7304186

[B12] Ceballos M. Llano Y. Salazar-Ospina A. Madrigal-Cadavid J. Pino-Marín D. Amariles P. (2021). Skills and practices of pharmacy staff for dispensing of drugs with fiscalized substances in drugstores and pharmacies. Rev. SaúdePública. 55, 44. 10.11606/s1518-8787.2021055003103 PMC8244815 34231824

[B13] Cheema E. Haseeb A. Khan T. M. Sutcliffe P. Singer D. R. (2017). Barriers to reporting of adverse drugs reactions: A cross sectional study among community pharmacists in United Kingdom. Pharm. Pract. (Granada) 15 (3), 931. 10.18549/PharmPract.2017.03.931 28943977 PMC5597805

[B14] Cheema E. Alhomoud F. K. Kinsara A. S. Alsiddik J. Barnawi M. H. Al-Muwallad M. A. (2018). The impact of pharmacists-led medicines reconciliation on healthcare outcomes in secondary care: A systematic review and meta-analysis of randomized controlled trials. PLoS One 13 (3), e0193510. 10.1371/journal.pone.0193510 29590146 PMC5873985

[B15] Emirates News Agency (2010). UAE has over 2000 private pharmacies, 4000 professionals. Available at URL: http://wam.ae/en/details/1395228626943.

[B16] Federation International Pharmaceutical (FIP) (2012). Pharmacy education taskforce a global competency framework. Version. Available from: PharmacyEducation/GbCF_v1.pdf (Accessed January 22, 2023).

[B17] Gokcekus L. Toklu H. Z. Demirdamar R. Gumusel B. (2012). Dispensing practice in the community pharmacies in the Turkish Republic of Northern Cyprus. Int. J. Clin. Pharm. 34, 312–324. 10.1007/s11096-011-9605-z 22262499

[B18] International Pharmaceutical Federation (2012). Pharmacy education taskforce: A global competency framework. Version 1. The Hague (NLD): FIP. Available from: https://www.fip.org/files/fip/PharmacyEducation/GbCF_v1.pdf.

[B19] Jairoun A. A. Al-Hemyari S. S. Jairoun M. El-Dahiyat F. Al-Ani M. Habeb M. (2021). Hidden factors in community pharmacy related to medication safety risks: pushing patient safety to breaking point. Res. Soc. Adm. Pharm. 16, S1551–S7411. 10.1016/j.sapharm.2021.11.005 34802959

[B20] Jairoun A. A. Al-Hemyari S. S. Shahwan M. Godman B. El-Dahiyat F. Kurdi A. (2022). Top unresolved ethical challenges and dilemmas faced by community pharmacists in providing pharmaceutical care: drawing the line between ethical challenges and the quality of the pharmaceutical care. Res. Soc. Adm. Pharm. 18, S1551–S7411. 10.1016/j.sapharm.2022.05.009 35618648

[B21] Kouladjian L. Chen T. F. Gnjidic D. Hilmer S. N. (2016). Education and assessment of pharmacists on the use of the drug burden index in older adults using a continuing professional development education method. Am. J. Pharm. Educ. 80 (4), 63. 10.5688/ajpe80463 27293230 PMC4891861

[B22] Lawshe C. (1975). A quantitative approach to content validity. Pers. Psychol. 28, 563–575. 10.1111/j.1744-6570.1975.tb01393.x

[B23] Mauricio C. Andrea S. Daniel S. Pedro A. (2022). Effectiveness of a continuing education program of drugs with fiscalized substance to improve pharmacy staff competencies: A multicenter, cluster-randomized controlled trial. Pharm. Pract. 20 (3), 2632. 10.18549/PharmPract.2022.3.2632 PMC9851815 36733513

[B24] Miotto K. Cho A. K. Khalil M. A. Blanco K. Sasaki J. D. Rawson R. (2017). Trends in tramadol: pharmacology, metabolism, and misuse. AnesthAnalg 124 (1), 44–51. 10.1213/ANE.0000000000001683 27861439

[B25] Palmer E. Hart S. Freeman P. R. (2017). Development and delivery of a pharmacist training program to increase naloxone access in Kentucky. J. Am. Pharm. Assoc. 57 (2S), S118–S122. 10.1016/j.japh.2016.12.071 28161300

[B26] Pervanas H. C. Revell N. Alotaibi A. F. (2016). Evaluation of medication errors in community pharmacy settings: A retrospective report. J. Pharm. Technol. 32 (2), 71–74. 10.1177/8755122515617199 34861023 PMC5998534

[B27] Polit D. F. Beck C. T. Owen S. V. (2007). Is the CVI an acceptable indicator of content validity? Appraisal and recommendations. Res. Nurs. Health 30 (4), 459–467. 10.1002/nur.20199 17654487

[B28] Preuss C. V. Kalava A. King K. C. (2019). “Prescription of controlled substances: benefits and risks,” in StatPearls [internet] (Treasure Island (FL): StatPearls Publishing). 30726003

[B29] Scahill S. L. Atif M. Babar Z. U. (2017). Defining pharmacy and its practice: A conceptual model for an international audience. Integr. Pharm. Res. Pract. 6, 121–129. 10.2147/IPRP.S124866 29354558 PMC5774311

[B30] Toklu H. Z. Akici A. Oktay Ş. Cali S. Sezen S. F. Keyer-Uysal M. (2010). The pharmacy practice of community pharmacists in Turkey. Marmara Pharm. J. 1 (14), 53–60. 10.12991/201014464

[B31] Vacca C. Orozco J. Figueras A. Capellà D. (2005). Assessment of risks related to medicine dispensing by nonprofessionals in Colombia: clinical case simulations. Ann. Pharmacother. 39 (3), 527–532. 10.1345/aph.1E420 15701777

[B32] Walters C. Raymont A. Galea S. Wheeler A. (2012). Evaluation of online training for the provision of opioid substitution treatment by community pharmacists in New Zealand. Drug Alcohol Rev. 31 (7), 903–910. 10.1111/j.1465-3362.2012.00459.x 22519647

[B33] Wheeler A. Fowler J. Hattingh L. (2013). Using an intervention mapping framework to develop an online mental health continuing education program for pharmacy staff. J. Contin. Educ. Heal Prof. 33 (4), 258–266. 10.1002/chp.21198 24347104

[B34] World Health Organization (WHO) (2012). Guide to estimate substance needs sometimes an international Audit. Available from: NarcoticDrugs/Guidelines/estimating_requirements/NAR_Guide_on_Estimating_SP_Ebook.pdf (Accessed December 3, 2022).

